# The History and Capabilities of Herbal Simples

**Published:** 1890-05-17

**Authors:** W. T. Fernie


### The History and Capabilities of Herbal Simples.

By W. T. Fernie, M.D.

(*Continued from page* 62.)

### IX.—BORAGE.

The herb Borage, with its gallant blue flower, is cultivated in our gardens, and is associated in our minds with bees and claret-cup. It grows wild in abundance on open plains where the soil is favourable; and it has a famous reputation for exhilarating the spirits. Botanically it is the Borago officinalis, as corrupted from corago, *i.e.*, cor, the heart; and ago, to act upon; quia cordis affectibus medetur. It was probably plentiful on the field of Waterloo, in June, 1813, when a regiment of Dutch soldiers, many thousands strong, turned tail as soon as the first shell fell some fifty yards off; and they fled bodily into Brussels, announcing the defeat of the allied forces, with the swift approach of the dreaded Napoleon. Who can tell but that a timely mess of borage-broth might have kindled fresh courage in their broad Dutch bosoms, and have made their bravery, instead of their cowardice, proverbial for ever? "Borago ego gaudia semper ago." I, Borage, bring always courage!

This plant was the Bugloss of the older botanists; and it corresponds to our common Bugloss, so called from the shape, and bristly surface of its leaves, which resemble Bous glossa, the rough tongue of the ox. Chemically the plant, which belongs to the natural order, Boragineæ, contains potassium and calcium, combined with mineral acids. The fresh juice affords thirty per cent. and the dried herb three per cent. of nitrate of potash. The stems and leaves supply much saline mucilage, which, when boiled and cooled, also deposits nitre and common salt. These saline ingredients promote activity of the kidneys: for which reasons the plant is much used in France as a remedy against feverish coughs and colds. The fresh herb has a slight cucumber-like odour;] and when compounded with lemon and sugar, and wine and water, it makes a delicious "cool tankard" for drinking in the summer. "A syrup concocted of the floures quieteth the lunatick person; and the leaves eaten raw do engender good blood." "Those of our time," says Gerarde, "do use the flowers in salads to make the heart glad. There be also many things made from the same for the comfort of the heart, to drive away sorrow, and to increase the joy of the mind." Old Burton tells us, in his Anatomy of Melancholy, that according to Dioscorides and Pliny, the borage was that famous nepenthe of Homer, which Polydamus sent to Helen for a token—"of such rare virtue that when taken steep't in wine, if wife and children, father and mother, brother and sister, and all thy dearest friends should die before thy face, thou could'st not grieve, or shed a tear for them." "The bowl of Helen had no other ingredient, as most criticks do conjecture, than this of borage." Parkinson avers that "the seed of borage helpeth nurses to have more store of milke, for which purpose its leaves are most conducing." Older writers declared of the herb "Vinum potatum quo sit macerata buglossa. Mœrorum cerebri dicunt auferre periti" ("to enliven the sad with the joy of a joke; Give them wine with some borage put in it to soak").

The flowers of the borage are of a vividly azure hue, which accounts for their finding such favour with the honey bee. Sir John Lubbock convinced himself by positive experiments that bees can distinguish different colours, and that they display a marked preference for blue. He placed some honey on several separate slips of glass, putting each slip over a piece of coloured paper, no two colours being alike. Then, having watched a bee make several journeys to a blue slip, he transposed this slip during an absence of the bee, and placed an orange slip where the blue slip had been, putting the blue slip at the same time about a foot away from the orange slip. The bee returned as usual to the place where she was accustomed to find the honey on the blue slip; but, though the honey was still there, seeing that it was not on a slip of blue colour, she did not alight, but paused for a moment, and then dashed straight away to the blue paper, a foot off. Also Sir John ranged in a row several slips of glass, each with honey on it, over papers of other colours, yellow, orange, red, green, black, and white, which were placed in close opposition to the blue slip; but, though he continually interchanged the relative arrangements of the various colours with regard to the blue slip, the bee always flew to the blue paper, wherever it might lie. It has been ascertained that as a rule, blue flowers are specialised for fertilisation by bees, who, therefore, prefer this colour; whilst conversely the flowers have acquired their blueness, because bees have shown a predilection for this hue. Mutually the flowers have become more and more blue; and the bees fonder and fonder of this attractive floral pigment, so as to promote their respective developments. Structurally out of the middle of the flowers grow forth black threads joined at the top like to a brooch, or pyramid. These black threads are the stamens, which stand like sentinels, in close order, around the pink pistil, to guard the yellow treasure-house of an ovary lying below. Of all honey-seeking insects, only bees know how to pronounce the "Open Sesame" which shall conciliate these stern soldiers of the black watch. For their prowess a guerdon of golden pollen besprinkles their bodies; and the scriptural injunction is given them—"That, whosoever shall next receive them, when they go out of that floral city, they shall shake off the fertilising dust from their feet as a testimony in its favour." A decoction or an infusion may be made for medicinal use from the stems, leaves, and flowers of the borage.

